# ^131^I-Caerin 1.1 and ^131^I-Caerin 1.9 for the treatment of non-small-cell lung cancer

**DOI:** 10.3389/fonc.2022.861206

**Published:** 2022-08-15

**Authors:** Na Liu, Tiantian He, Zewei Xiao, Juan Du, Keke Zhu, Xiongying Liu, Tongsheng Chen, Wenjuan Liu, Guoying Ni, Xiaosong Liu, Tianfang Wang, Jiangtao Quan, Jinhe Zhang, Peipei Zhang, Jianwei Yuan

**Affiliations:** ^1^ Department of Nuclear Medicine, The First Affiliated Hospital/Clinical Medical School, Guangdong Pharmaceutical University, Guangzhou, Guangdong, China; ^2^ Thoracic and Abdominal Radiotherapy Department, The First People’s Hospital of Foshan, Foshan, China; ^3^ Department of Nuclear Medicine, General Hospital of the Southern Theatre Command, People’s Liberation Army of China, Guangzhou, China; ^4^ Genecology Research Centre, University of the Sunshine Coast, Sunshine Coast, QLD, Australia

**Keywords:** high-affinity peptides, non-small-cell lung cancer, A549 cells, ^131^I labeling, Caerin peptide

## Abstract

**Objective:**

To investigate the effect of the ^131^I-labeled high-affinity peptides Caerin 1.1 and Caerin 1.9 for the treatment of A549 human NSCLC cells.

**Methods:**

**①** 3-[4,5-Dimethylthiazole-2-yl]-2,5-diphenyltetrazolium bromide (MTT) and plate clone formation assays were performed to confirm the *in vitro* anti-tumor activity of Caerin 1.1 and Caerin 1.9. ② Chloramine-T was used to label Caerin 1.1 and Caerin 1.9 with ^131^I, and the Cell Counting Kit 8 assay was performed to analyze the inhibitory effect of unlabeled Caerin 1.1, unlabeled Caerin 1.9, ^131^I-labeled Caerin 1.1, and ^131^I-labeled Caerin 1.9 on the proliferation of NSCLC cells. An A549 NSCLC nude mouse model was established to investigate the *in vivo* anti-tumor activity of unlabeled Caerin 1.1, unlabeled Caerin 1.9, ^131^I-labeled Caerin 1.1, and ^131^I-labeled Caerin 1.9.

**Results:**

**①** Caerin 1.1 and Caerin 1.9 inhibited the proliferation of NSCLC cells *in vitro* in a concentration-dependent manner. The half-maximal inhibitory concentration was 16.26 µg/ml and 17.46 µg/ml, respectively, with no significant intergroup difference (P>0.05). ② ^131^I-labeled Caerin 1.1 and ^131^I-labeled Caerin 1.9 were equally effective and were superior to their unlabeled versions in their ability to inhibit the proliferation and growth of NSCLC cells (P>0.05).

**Conclusions:**

^131^I-labeled Caerin 1.1 and ^131^I-labeled Caerin 1.9 inhibit the proliferation and growth of NSCLC cells and may become potential treatments for NSCLC.

## Introduction

The host defense polypeptides Caerin 1.1 (GLLSVLGSVALPHVLPHVVPVIAEHL-NH_2_) and Caerin 1.9 (GLFGVLGSIAKHVLPHVVPVIAEKL-NH_2_) are polypeptides derived from the gland secretions of Australian tree frogs (1). Over the past decades, several host defense peptides have been isolated and identified from the skin secretions of Australian tree frogs and toads. Many of these peptides have antimicrobial and/or neuropeptide activity (2). Some peptides are highly active against cancer cells (3). Caerin 1.1 and Caerin 1.9 have inhibitory effects on a variety of human tumor cells at a certain concentration but have no such effect on normal cells at the same concentration (4, 5).

The morbidity and mortality of cancer are rising rapidly worldwide (6), making it one of the major threats to human health. It is urgent to develop new treatments (7). In clinical practice, internal radiation therapy(IRT) refers to the introduction of radionuclides or their markers into the body, using the biological effects of ionizing radiation emitted by radionuclides α and β particles to inhibit or destroy tumor tissue, so as to achieve the purpose of treatment (8–10), while protecting normal tissues (11). For this procedure, radionuclides are conjugated with antibodies, antibody fragments, nanobodies, or small peptides, and the resulting high-affinity peptides bind specifically to the receptor on the cell surface or other highly expressed proteins on tumor cells (12). This method is an effective and promising treatment for cancer (13).

Lung cancer is the most common cancer worldwide and ranks first in cancer-related deaths the study of its biological mechanism has also become one of the hot issues (14). Non-small-cell lung cancer (NSCLC) accounts for more than 80% of lung cancer cases. Many cases are already advanced at the time of diagnosis (15, 16), therefore, NSCLC is characterized by high morbidity and mortality (17) and an adverse prognosis (18, 19). So, it is also necessary to find new treatments (20). While lung cancer may be treated with surgery, radiotherapy, immunotherapy, and chemotherapy (21), the 5-year survival rate remains low (less than 15%) (22). Therefore, it is important to develop new treatments that will improve survival.

In this study, we used chloramine-T to couple ^131^I with two high-affinity peptides (Caerin 1.1 and Caerin 1.9) and then analyzed the *in vivo* and *in vitro* effect of unlabeled Caerin 1.1, unlabeled Caerin 1.9, ^131^I-labeled Caerin 1.1, and ^131^I-labeled Caerin 1.9 for the treatment of NSCLC.

## Materials and methods

### Cell line and cell culture

The NSCLC cell line A549 was from the Stem Cell Bank of the Chinese Academy of Sciences. The medium of A549 cells contained 89% RPMI medium 1640 (GIBCO, USA), 10% heat-inactivated fetal bovine serum (Coring, USA), and 1% penicillin-streptomycin solution (GIBCO, USA). The cells were incubated in an incubator (Thermo, USA) at 37°C in 5% CO_2_.

### Peptides

The Caerin 1.1 polypeptide (GLLSVLGSVAKHVLPHVLPVVPVIAEHL-NH_2_) and the Caerin 1.9 polypeptide (GLFGVLGSIAKHVLPHVVPVIAEKL-NH_2_) were derived from the skin secretions of Australian tree frogs. The P3 polypeptide (GTELPSPPSVWFEAEF-OH) was used as the nonspecific control polypeptide. All three polypeptides were synthesized by China Polypeptide Limited (Shanghai, China), and their purity was >95% as determined by reversed-phase high-performance liquid chromatography. Caerin 1.1, Caerin 1.9, and P3 were dissolved in phosphate-buffered saline (PBS) (GIBCO, USA) to make solutions at different concentrations (10 mg/ml, 1 mg/ml, 0.1 mg/ml), which were stored at -20°C.

### Nude mouse

Specific pathogen–free (SPF) 4-6-week-old (adult) BALB/c female nude mice were purchased from Guangdong Medical Laboratory Animal Center. The animals were housed at the Animal Resource Center (First Affiliated Hospital of Guangdong Pharmaceutical University) under SPF conditions. No animal was sick or died during the study. Nude mice were sacrificed by cervical dislocation in accordance with the *Animal Management Measures* of the Ministry of Health of the People’s Republic of China (Document no.55, 2001). All experiments were approved and were done in accordance with the guidelines of the Animal Experiment Ethics Committee of the First Affiliated Hospital of Guangdong Pharmaceutical University (Ethics approval number: FAHGPU20160316).

### Effects of P3, Caerin 1.1, and Caerin 1.9 on the proliferation of A549 cells detected by MTT assay

A total of 500 mg of 3-(4,5-dimethylthiazol-2-yl)-2,5-diphenyl tetrazolium bromide (MTT) powder (Sigma-Aldrich, USA) was dissolved in 100 ml of PBS to a final concentration of 5 mg/ml (0.5% MTT). The solution was stored in the dark at -20°C. A549 cells in the exponential growth phase were seeded in 96-well plates (Coring, USA) at 5×10^3^ cells (100 µl) per well and then incubated at 37°C in 5% CO_2_ for 24 hours. Different concentrations of P3, Caerin 1.1, and Caerin 1.9 were added to the 96-well plates to reach final concentrations of 1, 5, 10, 15, 20, 25, 30, and 40 μg/ml. The experiment was done in triplicate and included control wells. The cells were cultured overnight. Once the drug’s effect was visible under the microscope, 10 µl of MTT solution was added to each well, and the cells were cultured for another 4 hours. Next, the supernatant was carefully aspirated, 150 µl of dimethyl sulfoxide solution (Sigma-Aldrich, USA) was added to each well, and the 96-well plates were placed on a shaker at low speed for 10 minutes. The absorbance at 570 nm was measured in an enzyme-linked immunosorbent assay plate reader (Thermo Scientific, USA). GraphPad Prism v9.3.0 was used calculate the half-maximal inhibitory concentration (IC_50_) of each drug and the survival rate (%).

### Plate clone formation assay

A549 cells were seeded into two six-well plates (Coring, USA) at 800 cells (2 ml) per well and then incubated overnight. On the next day, different concentrations of Caerin 1.1 and Caerin 1.9 were added to final concentrations of 0, 2.5, 5, 7.5, 10, and 15 µg/ml. The medium was changed every other day, and cell status and the number of clones observed. The cells were cultivated for 10 days, then fixed in 4% paraformaldehyde (Sigma, USA) for 20 minutes, stained with crystal violet solution (Beyotime Institute of Biotechnology, China) for 15 minutes, washed with PBS several times, and air-dried. ImageJ was used to count cell colonies, and GraphPad Prism v9.3.0 was used to compare groups.

### Preparation of ^131^I-labeled Caerin 1.1 and Caerin 1.9 (^131^I-Caerin 1.1, ^131^I-Caerin 1.9)

First, 50 mg of chloramine-T trihydrate (Shanghai Macklin Biochemical Co., Ltd., China) was dissolved in 10 ml of PBS at a final concentration of 5 mg/ml. The solution was stored at room temperature in the dark. Next, 40 µl of 1 μg/ml Caerin 1.1 or Caerin 1.9 was transferred to two 1.5-ml sterile Eppendorf tubes (Shanghai Hongsheng Technology, Co., Ltd., China), and 100 µl of 1 mCi(3.7×10^7^Bq) Na-^131^I solution (Guangdong Junqi Pharmaceutical Co., Ltd., China) and 100 µl of freshly prepared chloramine-T solution were added. The total reaction volume was 240 µl in each Eppendorf tube. A vortex mixer (Thermo Scientific, USA) was used to mix the mixture at room temperature for 10 minutes. Normal saline (Yangzhou Zhongbao Pharmaceutical Co., Ltd., China) was used as the developing agent, and the ^131^I-Caerin 1.1 and ^131^I-Caerin 1.9 labeling rates were determined by their radioactivity as measured with a γ counter (Zhongjia Optoelectronics Co., Ltd., China) and thin-layer paper chromatography. GraphPad Prism v9.3.0 was used to plot the γ count curve, which was used to calculate the labeling rate based on the area under the curve.


$$Labeling\;rate\;\left(\%\right)\;=\;\left({}^{131}I-Caerin\;1.1{/}^{131}I-Caerin\;1.9\;radioactive\;peak\;area\;integral\right)/total\;radioactive\;peak\;area\;integral$$

### Stability of ^131^I-Caerin 1.1 and ^131^I-Caerin 1.9

^131^I-Caerin 1.1 and ^131^I-Caerin 1.9 samples and their mixtures with fetal bovine serum and normal saline were stored at room temperature, 25°C, and 37°C. At different time points (0 h, 24 h, 72 h), the radiochemical purity (RCP) was measured with paper chromatography to determine the stability of the two labeled products in different solutions at different temperatures. GraphPad Prism v9.3.0 was used to calculate RCP and evaluate stability.

### Determination of lipid–water partition coefficient

A total of 800 µl of *n*-octanol (Shanghai Macklin Biochemical Co., Ltd., China), 800 µl of normal saline, and 50 µl of ^131^I-Caerin 1.1 or ^131^I-Caerin 1.9 were added to two different 1.5 ml Eppendorf tubes, which were sealed and placed on a shaker for 2 minutes and then centrifuged at 4000 rpm for 5 minutes to obtain the equilibrium state between *n*-octanol and normal saline. A sample of 50 µl was taken from the lipid phase and 50 µl from the aqueous phase, and the radioactivity count was determined for each tube. This experiment was repeated six times. The formula for the lipid–water partition coefficient (log P) was:


log P = log [(lipid phase γ count−background γ count)/(aqueous phase γ count−background γ count)]

### Cell uptake of ^131^I-Caerin 1.1 and ^131^I-Caerin 1.9

A549 cells were seeded in four 24-well plates (Coring, USA) at 5×10^4^ cells (500 µl) per well. The experiment was done in triplicate and included a positive-control well. After the cells were cultured in an incubator for 24 hours, the supernatant was discarded, 0.5 ml of serum-free medium was added to each well, and ^131^I-Caerin 1.1 or ^131^I-Caerin 1.9 and Na-^131^I solution (1.85 ×10^5^Bq or 2 µl per well) were added. After the cells were incubated for 2, 4, 6, or 24 hours, the cells were removed from the incubator. For the positive control, the supernatant was collected in a tube. For the experimental groups, the supernatant was discarded, and the cells were washed twice with PBS. Next, 200 µl of trypsin (GIBCO, USA) was added to each well. After digestion, the cells were washed three times with PBS and then collected in a tube to measure their radioactivity count. GraphPad Prism v9.3.0 was used to calculate the drug binding rate.

### Cell elution of ^131^I-Caerin 1.1 and ^131^I-Caerin 1.9

A549 cells were seeded in four 24-well plates at 5×10^4^ cells (500 µl) per well and cultured for 24 hours. The experiment was done in triplicate and included a positive-control well. The supernatant was discarded, 0.5 ml of serum-free medium was added to each well, and then ^131^I-Caerin 1.1, ^131^I-Caerin 1.9, or free ^131^I was added (1.85 ×10^5^Bq or 2 µl per well). After incubation for 24 hours, the supernatant was discarded, the cells were washed twice with PBS, 0.5 ml of serum-free medium was added to each well, and the cells were incubated for another 2, 4, 6, or 24 hours. For the positive control, the supernatant was collected in a tube. For the experimental groups, the supernatant was discarded, the cells were washed twice with PBS, and 200 µl of trypsin was added to each well. After digestion, the cells were washed three times with PBS and then collected in a tube to measure the radioactivity count. GraphPad Prism v9.3.0 was used to calculate the drug retention rate.

### Cell proliferation and cytotoxicity

A549 cells were seeded in two 96-well plates at 5×10^3^ cells (100 µl) per well and cultured for 24 hours at 5% CO_2_ and 37°C. The ^131^I-Caerin 1.1, ^131^I-Caerin 1.9, and Na-^131^I solutions were added to a final concentration of 2500, 5000, 10,000, or 20,000 KBq/ml. In the corresponding plates for Caerin 1.1 and Caerin 1.9, the drug concentration was 3.29, 6.95, 14.29, or 30.72 µg/ml. The experiment was done in triplicate and included three control (drug-free) groups. The cells were cultured for another 24 hours. Next, 10 µl of CCK-8 solution (DOJINDO, Japan) was added to each well, and then the cells were cultured in the dark for 4-6 hours. The absorbance at 450 nm was measured in an enzyme-linked immunosorbent assay plate reader, and GraphPad Prism v9.3.0 was used to calculate the cell survival rate.

### Interactions between ^131^I-Caerin 1.1 and ^131^I-Caerin 1.9

A549 cells were seeded in two 96-well plates at 5×10^3^ cells (100 µl) per well and cultured for 24 hours at 5% CO_2_ and 37°C. Add a gradient concentration of ^131^1-Caerin1.1 (0,2500,5000,10,000KBq/ml) and then ^131^I-Caerin1.9 of 0, 2500, 5000 and 10000 KBq/ml to each well. Pairwise pairing between the four concentrations of the two drugs requires a total of 16 groups with three compound wells each. After 24h, cell viability was assessed using the MTT assay.

### Tumorigenesis in animal models

A total of 100 µl of A549 cells (1×10^6^ cells) suspended in PBS were inoculated subcutaneously in the axillary area of nude mice to establish a subcutaneous xenograft model. Once the tumor was 3 to 4 mm long, the nude mice were used in the following experiment. A digital caliper (Mitutoyo, Japan, CD-15APX) was used to monitor tumor size every other day. Tumor volume was calculated according to the formula: volume = width^2^ × length/2.

To reduce the thyroid uptake of ^131^I, all nude mice were fed 0.1% potassium iodide (Shanghai Macklin Biochemical Co., Ltd., China) for 3 days to block the thyroid before the experiment. The A549 tumor-bearing nude mice were randomly divided into six groups of four to receive an intratumoral injection (100 µl) every other day for a total of four injections: PBS (control group), 8 μg of Caerin 1.1 in PBS (Caerin 1.1 group), 8 μg of Caerin 1.9 in PBS (Caerin 1.9 group), 7.4 ×10^6^ Bq Na-^131^I solution (^131^I group), 8 μg of ^131^I-Caerin 1.1 and 7.4 ×10^6^ Bq Na-^131^I solution (^131^I-Caerin 1.1 group), and 8 μg of ^131^I-Caerin 1.9 and 7.4 ×10^6^ Bq Na-^131^I solution (^131^I-Caerin 1.9 group). At 3 days after the last injection, the nude mice were sacrificed, and the tumors were isolated and weighed. GraphPad Prism v9.3.0 was used to analyze the body weight, tumor volume, and tumor weight.

### Statistical analysis

All experiments are repeated at least three times. GraphPad Prism v9.3.0 (San Diego CA, USA) was used for statistical analysis. The labeling rate was analyzed by Student’s t-test. Other data were analyzed by analysis of variance. P<0.05 was considered statistically significant.

## Results

### Caerin 1.1 and Caerin 1.9 inhibited the proliferation of A549 cells *in vitro*

**Figure 1:**
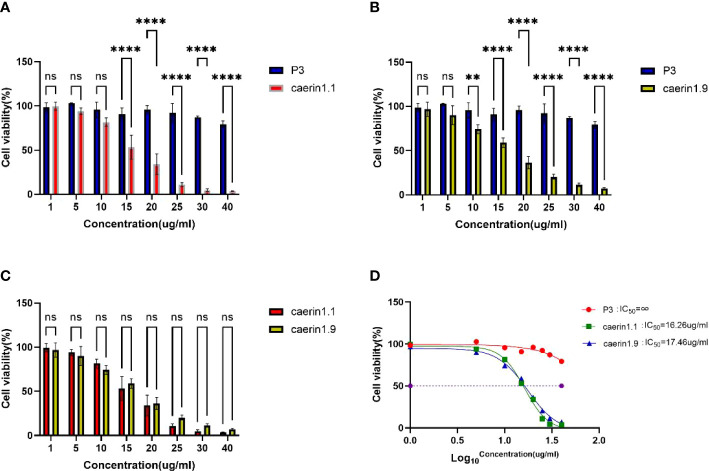
**(A–C)** Survival rate of A549 cells at different concentrations of P3, Caerin 1.1, and Caerin 1.9. **(D)** IC_50_ of P3, Caerin 1.1, and Caerin 1.9 on A549 cells. (NS: P>0.05; **: P<0.01; ****: P<0.0001).

Ma et al(2020) (3), Lin et al(2021) (5), Zhou et al(2020) (23), and Yuan et al(2018) (24) show that Caerin peptide inhibits the proliferation of cervical cancer cells, thyroid cancer cells, and breast cancer cells, without significant killing effects on a variety of mammalian cells (25–28). In this study, Caerin 1.1 and Caerin 1.9 had no significant inhibitory effect on A549 cells when their concentration was less than 5 μg/ml. At higher concentrations, Caerin 1.1 and Caerin 1.9 demonstrated strong inhibitory effects on the proliferation of A549 cells. As shown in **Figure 1**, the survival rate of A549 cells was 81.46% ± 3.52%, 53.32% ± 9.56%, and 34.12% ± 8.30% when the concentration of Caerin1.1 was 10 μg/ml, 15 μg/ml, and 20 μg/ml. When the concentration of Caerin1.9 was 10 μg/ml, 15 μg/ml, and 20 μg/ml, the survival rate of A549 cells was 74.20% ± 3.57%, 72.65% ± 0.76%, and 36.43% ± 4.85%, respectively. At 25 μg/ml, both Caerin 1.1 and Caerin 1.9 had significant inhibitory effects on the proliferation of A549 cells, and the cell survival rates were as low as 10.97% ± 1.55% and 20.43% ± 1.92%. As shown in **Figure 1C**, no significant difference was noted in the inhibitory effect between the two peptides at any concentration (P>0.05). In contrast, in the presence of high concentrations (30 µg/ml and 40 µg/ml) of nonspecific control peptide P3, the survival rate of A549 cells remained high (87.17% ± 0.95% and 79.26% ± 2.60%). As shown in **Figure 1D**, the IC_50_ was 16.26 µg/ml for Caerin 1.1 and 17.46 µg/ml for Caerin 1.9.

**Figure 2:**
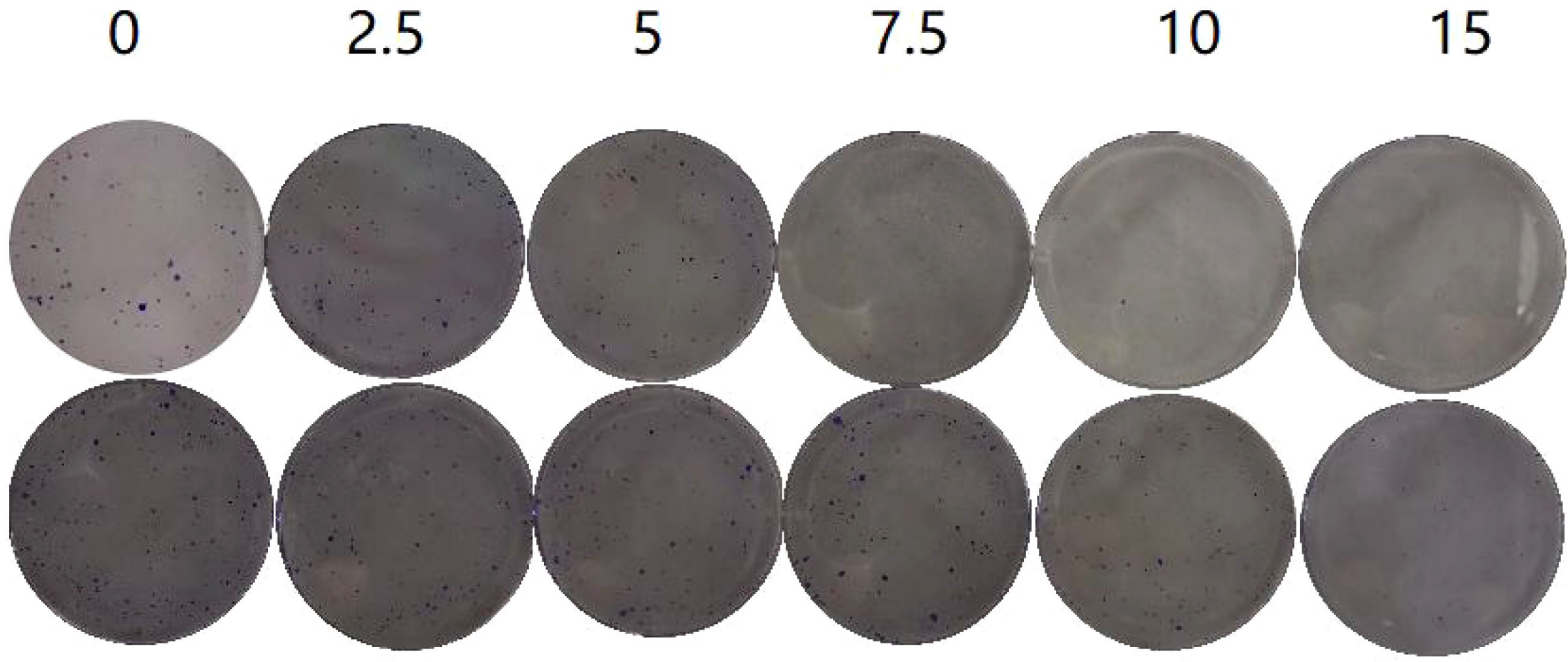
Plate clone formation assay to detect the number of A549 cell clones at different concentrations (0, 2.5, 5, 7.5, 10, 15 µg/ml) of Caerin 1.1 (**Figure 2**, top) and Caerin 1.9 (**Figure 2**, bottom).

**Figure 3:**
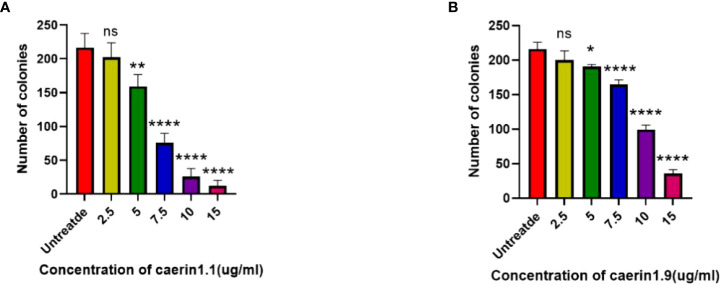
Number of cell clones at different concentrations of the drugs (control: 0 µg/ml). (NS: P>0.05; *: P<0.05; **: P<0.01; ****: P<0.0001).

In the plate clone formation assay (**Figures 2**, **3**), Caerin 1.1 and Caerin 1.9 had inhibitory effects on the proliferation of A549 cells that grew stronger with the concentration. Compared with the control group (0 µg/ml), a significant difference was noted in the number of cell clones when the concentration of the two peptides increased from 5 to 7.5 to 10 and then to 15 µg/ml (P<0.05). Their inhibitory effect on A549 cells seems to be concentration-dependent, with no significant difference between the two polypeptides.

### Labeling rate of ^131^I-Caerin 1.1 and ^131^I-Caerin 1.9

**Figure 4:**
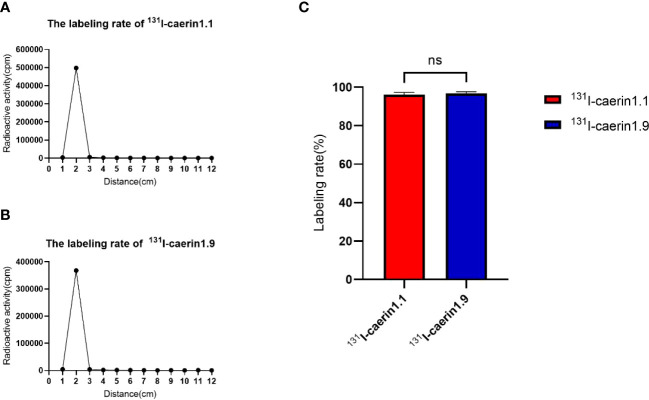
**(A, B)** The curves of the γ-counts of ^131^I-Caerin 1.1 and ^131^I-Caerin 1.9 with paper chromatography. The labeling rates were > 95%, with no significant difference between ^131^I-Caerin 1.1 and ^131^I-Caerin 1.9 **(C)**. (NS: P>0.05).

Chloramine-T was used to label Caerin 1.1 and Caerin 1.9 with ^131^I. The labeling rate was determined by paper chromatography. **Figure 4** shows that the radiolabeling rate was 96.20% ± 0.80% for ^131^I-Caerin 1.1 and 96.75% ± 0.66% for ^131^I-Caerin 1.9, with no significant difference between the two peptides (P>0.05).

### Stability of ^131^I-Caerin 1.1 and ^131^I-Caerin 1.9

**Figure 5:**
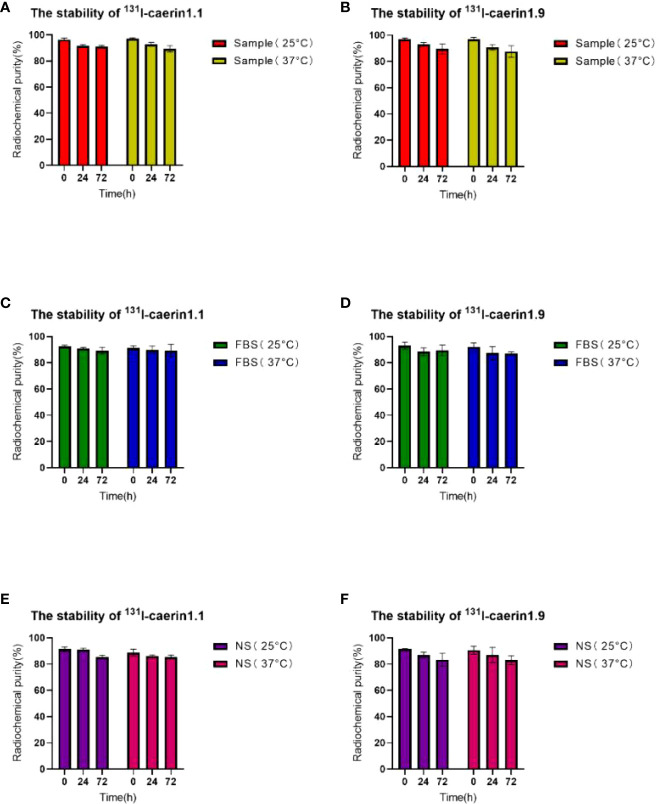
**(A, C, E)** RCP of 131I-Caerin 1.1 and their mixtures with FBS or NS at room temperature (25°C) or 37°C for different periods of time (0 h, 24 h, 72 h). **(B, D, F)** RCP of 131I-Caerin 1.9 and their mixtures with FBS or NS at room temperature (25°C) or 37°C for different periods of time (0 h, 24 h, 72 h).

**Figure 5** shows that ^131^I-Caerin 1.1 and ^131^I-Caerin 1.9 had a high RCP after 72 hours in fetal bovine serum (FBS) or normal saline (NS) at room temperature (25°C) or 37°C. The RCP was 91.06% ± 0.51%, 89.22% ± 1.87%, and 85.31% ± 1.01% for ^131^I-Caerin 1.1, ^131^I-Caerin 1.1 + FBS, and ^131^I-Caerin 1.1 + NS after 72 hours at 25°C; at 37°C, the numbers were 89.34% ± 1.69%, 89.22% ± 3.39%, and 85.43% ± 1.09%. For ^131^I-Caerin 1.9, the values were 89.51% ± 2.70%, 89.41% ± 2.87%, and 83.27% ± 3.46% at 25°C and 87.54% ± 3.11%, 87.13% ± 0.90%, and 82.99% ± 2.48% at 37°C. These data indicate that ^131^I-Caerin 1.1 and ^131^I-Caerin 1.9 and their mixtures with FBS or NS are stable at 25°C and 37°C.

### Lipid–water partition coefficient

LogP is an indicator of the solubility of the labeled product in the organic phase or the aqueous phase. LogP>0 indicates a trend towards fat solubility, and a higher LogP is associated with higher solubility in the organic phase (**Tables 1**, **2**). In this study, LogP=0.072 ± 0.007 (n=6) for ^131^I-Caerin 1.1 and 0.216 ± 0.040 (n=6) for ^131^I-Caerin 1.9, indicating that the prepared ^131^I-Caerin 1.1 and ^131^I-Caerin 1.9 were fat-soluble.

**Table 1:** Counts per minute (CPM) and lipid–water partition coefficient of ^131^I-Caerin 1.1 in the lipid phase and the aqueous phase.

No.	lipid phase γ count (CPM)	aqueous phase γ count (CPM)	LogP (mean ± SD)
1	2062596	1785234	
2	2045148	1770102	
3	2045082	1752036	
4	2021994	1690788	0.072 ± 0.007(n=6)
5	2007528	1662978	
6	1984464	1661442	
Mean	2027802	1720430	

**Table 2:** CPM and lipid–water partition coefficient of ^131^I-Caerin 1.9 in the lipid phase and the aqueous phase.

No.	lipid phase γ count (CPM)	aqueous phase γ count (CPM)	LogP (mean ± SD)
1	3672990	2403894	
2	3670302	2400372	
3	3620184	2372964	
4	3606900	2339928	0.216 ± 0.040(n=6)
5	3591120	1873314	
6	3486600	1866240	
Mean	3608016	2209452	

### Cell uptake and cell elution of ^131^I-Caerin 1.1 and ^131^I-Caerin 1.9

**Figure 6:**
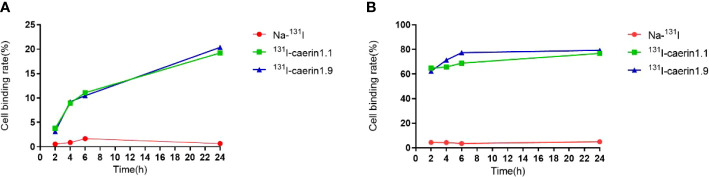
**(A)** Uptake rates of Na-^131^I, ^131^I-Caerin 1.1, and ^131^I-Caerin 1.9 by A549 cells at different time points (2 h, 4h, 6 h, 24 h); **(B)** Binding rates of Na-^131^I, ^131^I-Caerin 1.1, and ^131^I-Caerin 1.9 to A549 cells after incubation for up to 24 hours (2 h, 4h, 6 h, 24 h).

Cell uptake and cell elution experiments showed that both radiolabeled products were taken up and retained by A549 cells (**Figure 6**). The uptake of ^131^I-Caerin 1.1 and ^131^I-Caerin 1.9 by A549 cells increased with time. At 24 hours, the uptake rate was 19.21% ± 1.49% for ^131^I-Caerin 1.1 and 20.37% ± 1.18% for ^131^I-Caerin 1.9. No significant uptake of Na-^131^I was noted (0.67 ± 0.08% at 24 h).

In the cell elution experiment, the two drugs were incubated with A549 cells for up to 24 hours before elution. The binding rate remained high at each time point and increased slightly over time (2h, 4h, 6h, 24h). At 24 hours, the binding rate was 76.77% ± 11.69% for ^131^I-Caerin 1.1, 79.30% ± 7.08% for ^131^I-Caerin 1.9, and 4.97% ± 0.59% for Na-^131^I.

### Cell proliferation and cytotoxicity

**Figure 7:**
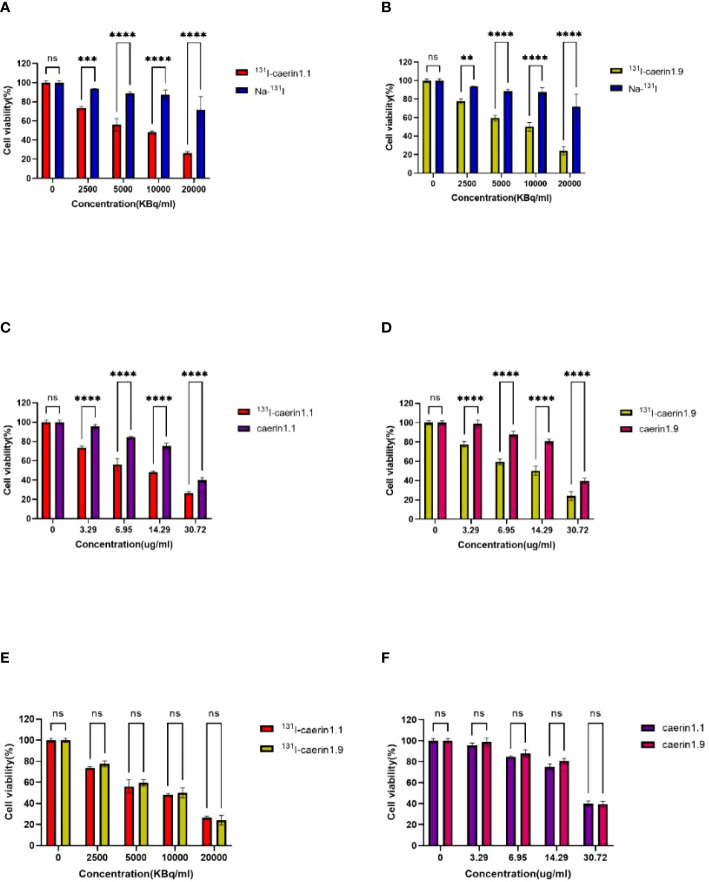
**(A, B)** Survival rate of A549 cells at different concentrations (2500, 5000, 10,000, 20,000 KBq/ml) of ^131^I-Caerin 1.1, ^131^I-Caerin 1.9, and Na-^131^I. **(C, D)** Survival rate of A549 cells at different concentrations (3.29, 6.95, 14.29, 30.72 µg/ml) of ^131^I-Caerin 1.1 versus Caerin 1.1 and ^131^I-Caerin 1.9 versus Caerin 1.9. **(E, F)** Survival rate of A549 cells at different concentrations of ^131^I-Caerin 1.1 versus ^131^I-Caerin 1.9 and at different concentrations of Caerin 1.1 versus Caerin 1.9. (NS: P>0.05; **: P<0.01; ***: P<0.001; ****: P<0.0001).

The CCK-8 assay (**Figure 7**) showed that the level of cytotoxicity increased and the cell survival rate decreased with higher concentrations of ^131^I-Caerin 1.1 and ^131^I-Caerin 1.9. For ^131^I-Caerin 1.1, at 2500, 5000, 10,000, and 20,000 KBq/ml, the survival rates of A549 cells were 73.62% ± 1.04%, 56.02% ± 4.50%, 48.27% ± 0.87%, and 26.49% ± 1.13%; at the equivalent concentrations (3.29, 6, 95, 14.29, 30.72 µg/ml) of Caerin 1.1, the inhibitory effect on A549 cells was significantly weaker than that of ^131^I-Caerin 1.1 (P<0.05). For ^131^I-Caerin 1.9, at 2500, 5000, 10,000, and 20,000 KBq/ml, the survival rates of A549 cells were 77.65% ± 1.99%, 59.48% ± 2.13%, 50.20% ± 3.27%, and 24.13% ± 3.13%; at the equivalent concentrations of Caerin 1.9, the inhibitory effect on A549 cells was significantly weaker (P<0.05). Na-^131^I showed an inhibitory effect on A549 cell proliferation only at a high concentration (20,000 KBq/ml), with a cell survival rate of 71.56% ± 9.83%. **Figure 7E** shows that the levels of cytotoxicity of ^131^I-Caerin 1.1 and ^131^I-Caerin 1.9 on A549 cells remained largely unchanged across different concentrations (P>0.05). **Figure 7F** is consistent with the MTT assay results, where no significant difference was noted in the inhibitory effect of different concentrations of Caerin 1.1 and Caerin 1.9 on the proliferation of A549 cells (P>0.05).

### Interactions between ^131^I-Caerin 1.1 and ^131^I-Caerin 1.9

The synergy score was calculated using the SynergyFinder software and resulted in 0.082.

When synergy score is from -10 to 10: the interaction between two drugs is likely to be additive, so the interaction between ^131^I-Caerin 1.1 and ^131^I-Caerin 1.9 is likely to be additive.

### ^131^I-Caerin 1.1 and ^131^I-Caerin 1.9 inhibit the growth of xenograft NSCLC tumors in nude mice

**Figure 8:**
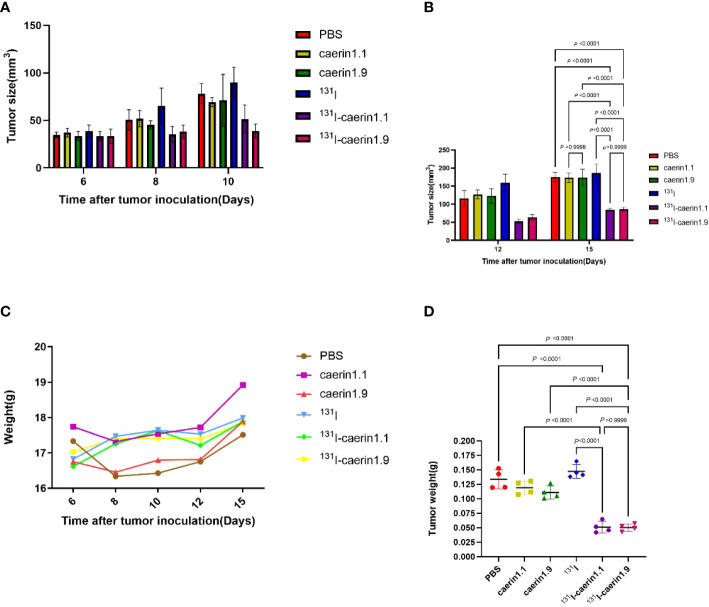
**(A, B)** Tumor volume at different time points after tumor graft in different groups. **(C)** Body weight of nude mice at different time points after tumor graft in different groups. **(D)** Tumor weight in different groups.

**Figure 9:**
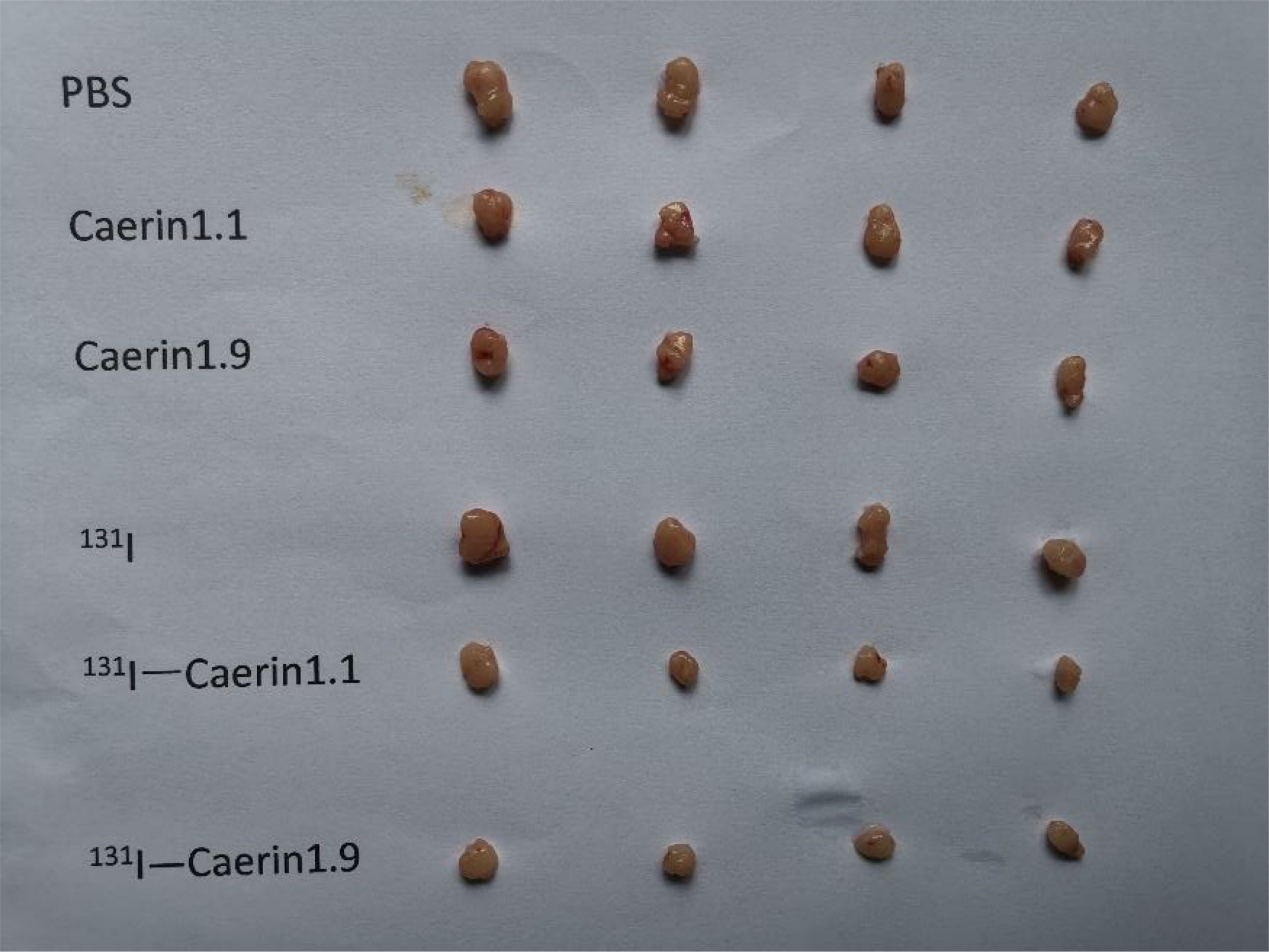
Pictures of the tumors isolated from different groups.

Next, we investigated whether ^131^I-Caerin 1.1 and ^131^I-Caerin 1.9 inhibit the growth of NSCLC *in vivo*. No significant between-group difference was noted in tumor volume before treatment (P>0.05). **Figure 8C** shows that the body weight of nude mice increased in each group after treatment. At the time of sacrifice, the body weight was (17.51 ± 0.32) g in the PBS group, (18.92 ± 0.68) g in the Caerin 1.1 group, (17.89 ± 0.56) g in the Caerin 1.9 group, (17.99 ± 0.50) g in the ^131^I group, (17.89 ± 0.56) g in the ^131^I-Caerin 1.1 group, and (17.83 ± 0.28) g in the ^131^I-Caerin 1.9 group. **Figures 8A, B** show that the tumor volume increased in each group after treatment, but the tumor grew significantly slower in the ^131^I-Caerin 1.1 group and the ^131^I-Caerin 1.9 group than in the PBS group, the ^131^I group, the Caerin 1.1 group, or the Caerin 1.9 group. At the time of sacrifice, the tumor volume was (175.22 ± 9.88) mm^3^ in the PBS group, (173.18 ± 9.93) mm^3^ in the Caerin 1.1 group, (173.90 ± 18.00) mm^3^ in the Caerin 1.9 group, (186.30 ± 19.69) mm^3^ in the ^131^I group, (84.98 ± 2.71) mm^3^ in the ^131^I-Caerin 1.1 group, and (86.66 ± 3.90) mm^3^ in the ^131^I-Caerin 1.9 group. After sacrifice, the tumors were isolated and weighed. Their weights were (0.134 ± 0.013) g in the PBS group, (0.119 ± 0.009) g in the Caerin 1.1 group, (0.111 ± 0.009) g in the Caerin 1.9 group, (0.147 ± 0.009) g in the ^131^I group, (0.051 ± 0.008) g in the ^131^I-Caerin 1.1 group, and (0.050 ± 0.005) g in the ^131^I-Caerin 1.9 group. These data indicate that ^131^I-Caerin 1.1 and ^131^I-Caerin 1.9 inhibit the growth of NSCLC *in vivo*. At the end of treatment, significant differences were noted in both tumor volume and tumor weight between the PBS group, the ^131^I group, the Caerin 1.1 group, and the Caerin 1.9 group (P<0.05). These data are consistent with the CCK-8 assay results. No significant difference was noted in tumor volume or tumor weight between the ^131^I-Caerin 1.1 group and the ^131^I-Caerin 1.9 group (P>0.05). Pictures of the isolated tumor samples are shown in **Figure 9**.

## Discussion

Lung cancer is usually treated with surgery, chemotherapy, radiotherapy, anti-angiogenesis inhibitors, and tyrosine kinase inhibitors. The overall response rate and the survival rate remain low for NSCLC, so new drugs and combination therapies are needed in order to improve the prognosis of NSCLC (29).

During IRT with radionuclides, radionuclides or their labeled products are introduced into the body (30), and the biological effects of α and β particles emitted by the radionuclides ultimately result in the death of cancer cells. In this study, the MTT assay showed that both Caerin 1.1 and Caerin 1.9 inhibited the proliferation of NSCLC cells (A549) *in vitro* in a concentration-dependent manner. Their IC_50_ were 15.62 µg/ml and 18.52 µg/m, respectively (P>0.05). Zhou et al(2020) (23) showed that Caerin 1.1 inhibits papillary thyroid cancer cells (B-CPAP cells) and anaplastic thyroid cancer cells (CAL-62 cells) with an IC_50_ value of 4.038 μg/ml and 9.856 μg/ml, respectively. Caerin 1.1 has its highest IC_50_ for A549 cells and lowest for B-CPAP cells, which may be associated with the relatively low degree of malignancy of papillary thyroid carcinoma (31). Here, the plate clone formation assay confirmed that the inhibitory effect of Caerin 1.1 and Caerin 1.9 on the proliferation of NSCLC (A549) cells increased with the drug concentration, with no significant difference between the two peptides (P>0.05). Lin et al. (2021) (5) concluded that in cancer cells, a potential mechanism of action of Caerin 1.1 is to inactivate the phosphoinositide 3-kinase (PI3K)/protein kinase B (Akt) pathway and inhibit its overexpression or its mutant signal components, thereby promoting cell apoptosis. Ni et al. (2018) (32) showed that both Caerin 1.1 and Caerin 1.9 induce TC-1 cell apoptosis *in vitro* by different mechanisms. Instead of promoting normal apoptosis, they may target tumors *in vivo* to alter the tumor microenvironment, thereby suppressing the tumor growth. In this study, we focused on how Caerin 1.1 and Caerin 1.9 inhibit the growth of NSCLC.

^131^I (half-life: 8.04 days) is the most common radioisotope used in radionuclide therapy (23). Based on the principle of IRT with radionuclides, we used chloramine-T to prepare two ^131^I-labeled products, including ^131^I-Caerin 1.1 and ^131^I-Caerin 1.9, achieving labeling rates greater than 95%. Moreover, the labeled products were stable in NS and FBS at different temperatures (25°C and 37°C), which was a good foundation for subsequent experiments. The lipid–water partition coefficient showed that both labeled products are fat-soluble, indicating that they may be excreted mainly through the liver. In cell uptake and cell elution experiments, the Na-^131^I group was used as the control group for ^131^I-Caerin 1.1 and ^131^I-Caerin 1.9. The results showed that the binding rates of ^131^I-Caerin 1.1 and ^131^I-Caerin 1.9 to A549 cells increased over time and were significantly higher than that of Na-^131^I, indicating that ^131^I-Caerin 1.1 and ^131^I-Caerin 1.9 are taken up by NSCLC (A549) cells. In the absence of sodium/iodide symporter (NIS) (22), NSCLC (A549) cells take up very little Na-^131^I, consistent with our results.

Our cell elution experiment showed high retention rates (70-80%) of ^131^I-Caerin 1.1 and ^131^I-Caerin 1.9 in A540 cells at 2, 4, 6, and 24 hours. MTT assays have shown that Caerin 1.1 is taken up by CAL-62 and B-CPAP cells and is mainly enriched in the cytoplasm (23), suggesting that Caerin 1.1 and Caerin 1.9 can also be taken up by NSCLC (A549) cells to exert an inhibitory effect. Moreover, ^131^I can enter A549 cells, leading to iodine accumulation in NSCLC (A549) cells despite the lack of a NIS, then enhanced proliferative toxic effect on A549 cells. Drug interaction experiments have shown that the combination of ^131^I-Caerin1.1 and ^131^I-Caerin1.9 may be additive, providing a certain basis for the subsequent joint application of the two drugs.

*In vivo*, we successfully established a nude mouse model of xenograft NSCLC (A549) cells. The results showed that tumor volume and tumor weight were significantly lower in the ^131^I-Caerin 1.1 group and the ^131^I-Caerin 1.9 group than in the control group (the PBS group), the peptide groups (the Caerin 1.1 group, the Caerin 1.9 group), and the ^131^I group (P<0.05), without a significant difference between the ^131^I-Caerin 1.1 group and the ^131^I-Caerin 1.9 group (P>0.05). Before choosing the dose of the drug injection, we measured the labeling rate of different concentrations of Caerin 1.1 or Caerin 1.9 with 3.7 ×10^7^ Bq ^131^I. The results of repeat experiments showed that the labeling rate was highest when the concentration of Caerin 1.1 or Caerin 1.9 was 40 µg, which was used for subsequent experiments. When 7.4 ×10^6^ Bq Na-^131^I solution was injected into each nude mouse in the ^131^I group, 8 μg of Caerin 1. 1 or Caerin 1.9 and 7.4 ×10^6^ Bq Na-^131^I solution were injected into each mouse in the ^131^I-Caerin 1.1 group or the ^131^I-Caerin 1.9 group. For the Caerin 1.1 group and the Caerin 1.9 group, only 8 µg of Caerin 1.1 or Caerin 1.9 was injected. At the end of treatment, no significant difference was noted in tumor volume or tumor weight between the Caerin 1.1 group, the Caerin 1.9 group, the PBS group, and the ^131^I group (P>0.05), which contrasts with our previous *in vitro* experiments (MTT assay, plate clone formation). This may be because the amount of peptide used in the Caerin 1.1 group and the Caerin 1.9 group was too low to have any significant inhibitory effect. In the future, we will investigate the inhibitory effects of different doses of peptides on tumor growth *in vitro*. NSCLC (A549) cells do not express NIS; as a result, Na-^131^I does not enter NSCLC (A549) cells or exert any significant inhibitory effect on these cells. This is consistent with our *in vitro* inhibition experiment.

Both *in vitro* (CCK-8 assay) and *in vivo* experiments showed that ^131^I-Caerin 1.1 and ^131^I-Caerin 1.9 had stronger inhibitory effects on NSCLC cells than Caerin 1.1 and Caerin 1.9. These data, along with the cell uptake data, show that the two peptides can carry ^131^I into cells, leading to iodine accumulation in NSCLC (A549) cells despite their lack of a NIS. Once in the cells, ^131^I-Caerin 1.1 and ^131^I-Caerin 1.9 exert both the biological effects of ^131^I and their intrinsic anti-tumor effects, which synergistically enhance their inhibitory effect on NSCLC.

Caerin 1.1 and Caerin 1.9, and especially ^131^I-Caerin 1.1 and ^131^I-Caerin 1.9, inhibit the growth of NSCLC (A549) cells *in vitro* in a concentration-dependent manner. Animal experiments show that ^131^I-Caerin 1.1 and ^131^I-Caerin 1.9 inhibit the growth of NSCLC *in vivo*. No significant difference is noted in the inhibitory effect on NSCLC (A549) cells between Caerin 1.1 and Caerin 1.9, the labeling rate between ^131^I-Caerin 1.1 and ^131^I-Caerin 1.9, or the *in vivo* or *in vitro* inhibitory effect on A549 cells between ^131^I-Caerin 1.1 and ^131^I-Caerin 1.9 (P>0.05). This may be related to the similar functional high-affinity amino acids contained in Caerin 1.1 and Caerin 1.9.

The next thing we need to do is to assess the toxicity of ^131^ I-Caerin 1.1 and ^131^ I-Caerin1.9 and its biodistribution in different organs using histological and biochemical assays.

For evaluating toxicity of ^131^I-caerin1.1 and ^131^I-caerin1.9, tumor-bearing mice were killed after mice were injected with ^131^I-caerin1.1 or ^131^I-caerin1.9 and sacrificed after 3 days. Their blood was collected and plasma was discarded for concentration analyzes of alanine aminase (ALT), aspartate transaminase (AST), blood urea nitrogen (BUN) and creatinine (Crea)alanine ransaminase (ALT), aspartate transaminase (AST), blood urea nitrogen (BUN), creatinine (CREA). Meanwhile, organs of brain, lung, spleen, kidney and liver were collected, fixed, paraffin embedded and post-stained for histopathological examination (33–35).

Tumor-bearing mice were killed by an overdose of pentobarbital sodium 24h after injected with ^131^I-caerin1.1 or ^131^I-caerin1.9, then tumor, kidney, liver, brain, lung and spleen were collected to measure radioactive counts within different organs to reflect the biological distribution of ^131^I-caerin1.1 and ^131^I-caerin1.9 in different organs (36).

## Conclusion

Despite different peptide sequences, Caerin 1.1 and Caerin 1.9 and their labeled products (^131^I-Caerin 1.1 and ^131^I-Caerin 1.9) share similar physical and chemical properties, with similar inhibitory effects on the proliferation of NSCLC cells. ^131^I-Caerin 1.1 and ^131^I-Caerin 1.9 have stronger inhibitory effects on xenogeneic NSCLC tumors in nude mice than unlabeled Caerin 1.9 and Caerin 1.1, indicating that ^131^I-Caerin 1.1 and ^131^I-Caerin 1.9 may become potential treatments for NSCLC.

## Data availability statement

The original contributions presented in the study are included in the article/Supplementary Material. Further inquiries can be directed to the corresponding authors.

## Ethics statement

The animal study was reviewed and approved by The Animal Experiment Ethics Committee of the First Affiliated Hospital of Guangdong Pharmaceutical University (Ethics approval number: FAHGPU20160316).

## Author contributions

NL, JY, XSL, TW and GN contributed to the study’s conception and design. Material preparation, experimental operation was performed by NL, TH, ZX, KZ and JD. Date processing and analysis was carried out by XYL, TC and WL. The statistical methods were reviewed by JQ and JZ. The first draft of the manuscript was written by NL and was revised by JY and PZ. All authors contributed to the article and approved the submitted version.

## Funding

This study was funded by the National Natural Science Foundation (No. 31971355).

## Conflict of interest

The authors declare that the research was conducted in the absence of any commercial or financial relationships that could be construed as a potential conflict of interest.

## Publisher’s note

All claims expressed in this article are solely those of the authors and do not necessarily represent those of their affiliated organizations, or those of the publisher, the editors and the reviewers. Any product that may be evaluated in this article, or claim that may be made by its manufacturer, is not guaranteed or endorsed by the publisher.
